# Hand Osteomyelitis: Exploratory Associations of Admission C-Reactive Protein and MRI with Surgical Burden and Hospital Length of Stay

**DOI:** 10.3390/jcm15176729

**Published:** 2026-08-30

**Authors:** Jessica Köller, Alberto Alfieri Zellner, Jonas Roos, Johanna Thiele, Lisa Fiona Roder, Mari Babasiz, Kristian Welle, Christian Prangenberg

**Affiliations:** Department of Orthopedics and Trauma Surgery, University Hospital Bonn, 53127 Bonn, Germany

**Keywords:** hand osteomyelitis, magnetic resonance imaging, finger amputation

## Abstract

**Background:** Hand osteomyelitis is an uncommon but potentially devastating infection associated with prolonged treatment, repeated surgical interventions, and risk of amputation. Owing to its rarity, evidence regarding prognostic factors and optimal management remains limited. This study aimed to evaluate the clinical characteristics, microbiological findings, treatment strategies, and outcomes of patients with hand osteomyelitis, with particular emphasis on the association of admission inflammatory markers, immunosuppression, and preoperative magnetic resonance imaging (MRI). **Methods:** A retrospective single-center cohort study was performed over a 11-year period at a tertiary referral hospital. Patients with clinically and/or histopathologically confirmed hand osteomyelitis were identified using ICD-10-GM codes. Demographic characteristics, etiology, microbiological and histopathological findings, laboratory parameters, imaging, surgical treatment, antibiotic therapy, and length of hospital stay were reviewed. Correlations between admission C-reactive protein (CRP), leukocyte count, number of surgical procedures, and hospital stay were analyzed using Pearson’s correlation coefficient. **Results:** Fourteen patients were included (mean age, 53.7 years; 64.3% female). Osteomyelitis predominantly affected the distal phalanx (92.9%). Immunosuppression or immunocompromising comorbidities were present in 64.3% of patients. Staphylococcus aureus, methicillin-resistant Staphylococcus aureus, and Streptococcus pyogenes were the most frequently isolated pathogens, while immunosuppressed patients exhibited more polymicrobial infections. The mean admission CRP was 87.8 mg/L and was markedly higher in immunosuppressed than in non-immunosuppressed patients (128.5 vs. 14.6 mg/L). Amputation was required in 78.6% of patients, with a mean of 3.3 surgical procedures per patient. Admission CRP is associated with both length of hospital stay (r = 0.61) and number of surgical procedures (r = 0.73), whereas leukocyte count showed only weak correlations. Patients who underwent MRI had a shorter mean hospital stay than those without MRI (13.4 vs. 19.6 days). The mean duration of antibiotic therapy was 29.3 days. **Conclusions:** In this small cohort, admission CRP showed a potential association with disease severity in hand osteomyelitis, with higher levels observed in patients requiring more extensive surgical treatment and longer hospitalization. MRI was associated with a shorter hospital stay, suggesting a possible role of early advanced imaging in facilitating timely diagnosis and treatment planning. Immunosuppressed patients tended to exhibit higher inflammatory markers and more frequently polymicrobial infections, although these findings should be interpreted cautiously given the limited sample size. Overall, these observations are exploratory and require confirmation in larger, preferably prospective studies.

## 1. Introduction

Osteomyelitis of the hand is an uncommon but potentially devastating infection that may result in irreversible bone destruction, loss of function, and amputation if diagnosis and treatment are delayed. Owing to its low incidence, current evidence is largely limited to retrospective case series, and standardized diagnostic and therapeutic algorithms remain lacking [1,2].

Unlike osteomyelitis of long bones, hand osteomyelitis most commonly develops following direct inoculation through penetrating trauma, bite injuries, postoperative infections, or contiguous spread from soft tissue infections. Hematogenous dissemination is comparatively rare in adults [3,4].

The microbiological spectrum is broad, although *Staphylococcus aureus* remains the predominant pathogen. Polymicrobial infections are frequently encountered in immunocompromised patients, patients with diabetes mellitus or peripheral vascular disease, and following human or animal bites, necessitating broad empirical antimicrobial coverage until culture results become available [1,5,6].

**Figure 1:**
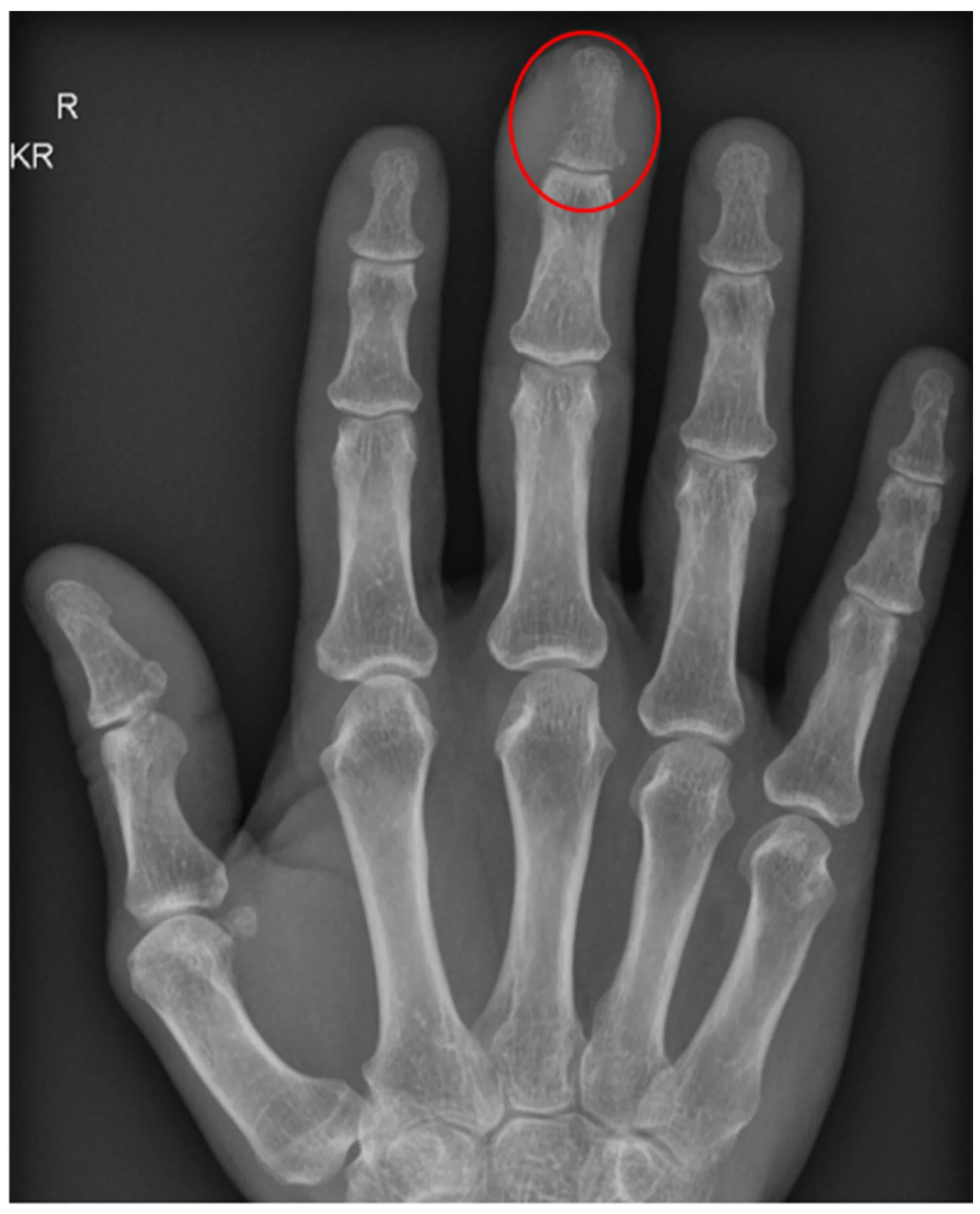
Anteroposterior radiograph of the right hand. The circled area demonstrates osteolytic bone destruction of the distal phalanx of the middle finger (digit III), consistent with distal phalangeal osteomyelitis.

**Figure 2:**
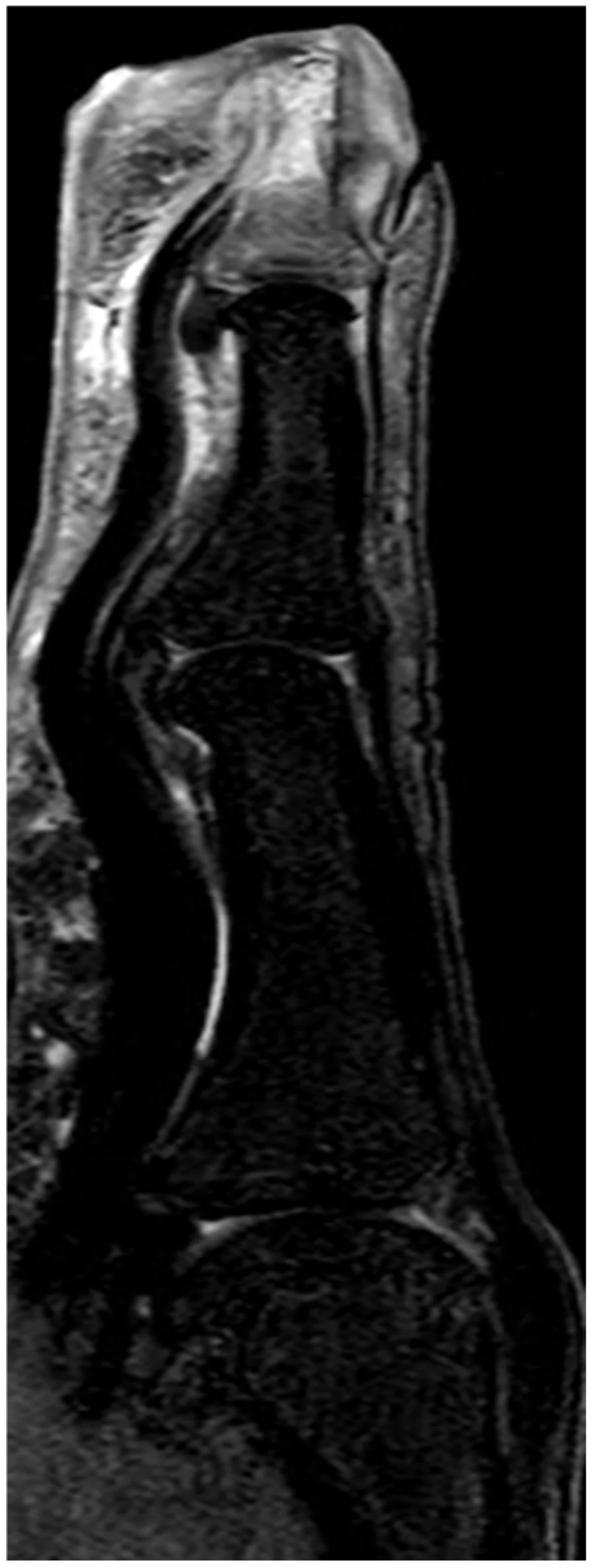
Sagittal T2-weighted magnetic resonance image (MRI) of the middle finger (digit III) demonstrating bone marrow edema and inflammatory changes in the distal phalanx, consistent with distal phalangeal osteomyelitis.

Early diagnosis remains challenging because clinical symptoms are often nonspecific and conventional radiographs may appear normal during the initial stages of infection. Magnetic resonance imaging (MRI) is considered the most sensitive imaging modality for detecting early bone marrow edema and defining the extent of infection, thereby facilitating timely surgical planning [1,7,8] (Figure 1 and Figure 2).

Inflammatory biomarkers, particularly C-reactive protein (CRP), are routinely used in the diagnostic work-up and follow-up of musculoskeletal infections. Although neither CRP nor leukocyte count is sufficiently specific to establish the diagnosis of osteomyelitis, elevated CRP levels have been associated with disease severity and treatment outcomes in several studies [1,9].

The cornerstone of treatment consists of prompt surgical debridement combined with targeted antimicrobial therapy based on microbiological cultures. Recent evidence has demonstrated that, following adequate surgical management, an early switch from intravenous to oral antibiotic therapy is non-inferior to prolonged intravenous treatment for most bone and joint infections [1,10].

Despite advances in imaging, microbiological diagnostics, and antimicrobial therapy, delayed diagnosis continues to be associated with prolonged hospitalization, repeated surgical interventions, and an increased risk of amputation, particularly in patients with significant comorbidities or immunosuppression [1,4].

The aim of the present study was to characterize the clinical presentation, microbiological findings, treatment strategies, and outcomes of patients with hand osteomyelitis treated at a tertiary referral center over a 10-year period. In particular, we investigated whether admission inflammatory markers, immunosuppression, and preoperative MRI were associated with disease severity, surgical burden, and length of hospital stay.

## 2. Materials and Methods

**Figure 3:**
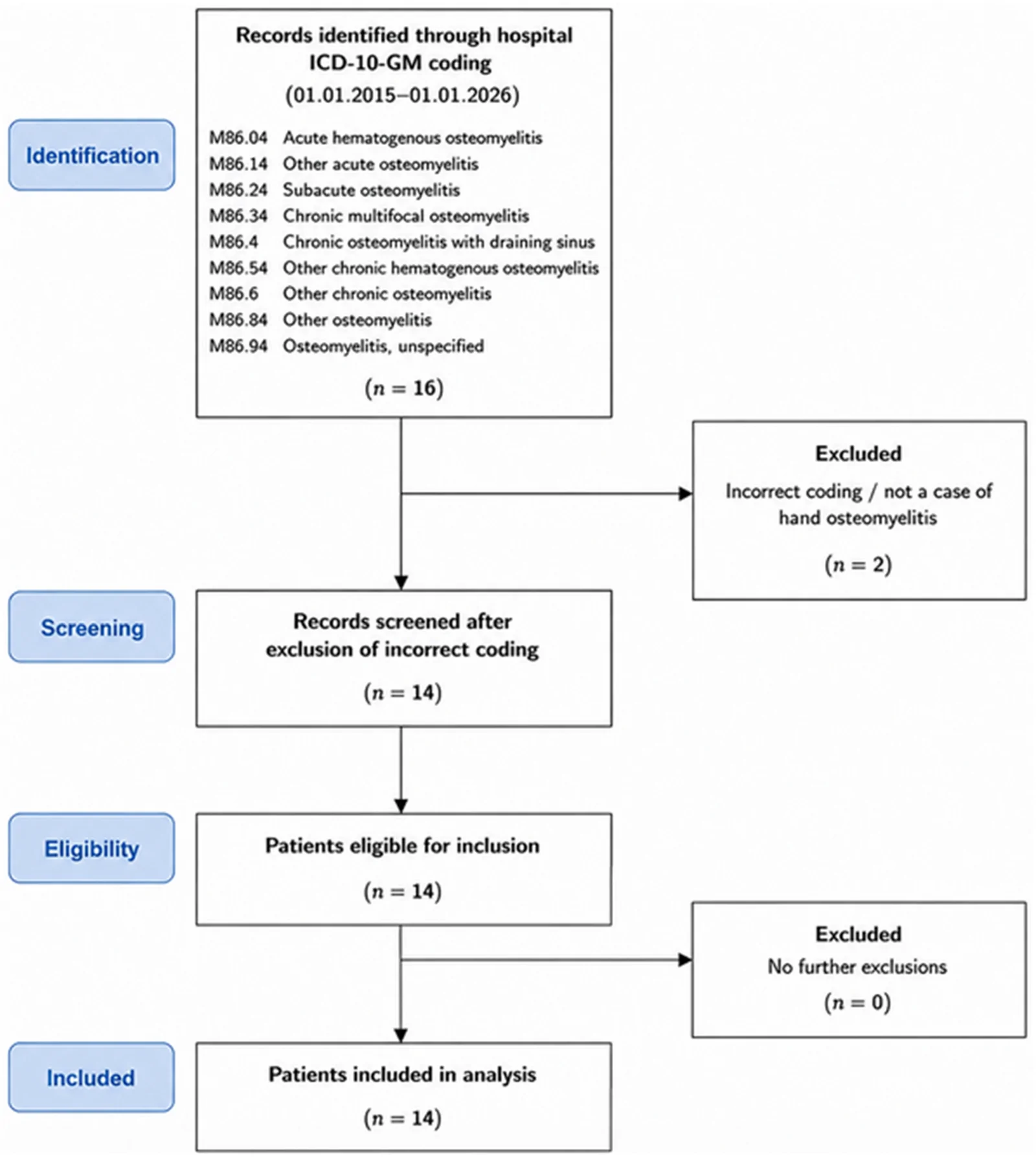
STROBE flow diagram of patient identification and inclusion. Patients with hand osteomyelitis were identified using ICD-10-GM codes between 1 January 2015 and 1 January 2026. Of 16 initially identified patients, two were excluded due to incorrect coding, resulting in 14 patients included in the final analysis.

A retrospective single-center cohort study was conducted at a tertiary referral hospital. The institutional hospital information system was screened over a 11-year period for patients diagnosed with osteomyelitis of the hand (1 January 2015–1 January 2026). Eligible cases were identified using the International Classification of Diseases, 10th Revision, German Modification (ICD-10-GM) codes for osteomyelitis, including M86.04 (acute hematogenous osteomyelitis), M86.14 (other acute osteomyelitis), M86.24 (subacute osteomyelitis), M86.34 (chronic multifocal osteomyelitis), M86.4 (chronic osteomyelitis with draining sinus), M86.54 (other chronic hematogenous osteomyelitis), M86.6 (other chronic osteomyelitis), M86.84 (other osteomyelitis), and M86.94 (osteomyelitis, unspecified). (Figure 3).

The diagnosis of hand osteomyelitis was established by the treating physicians based on the overall clinical, intraoperative, microbiological, and histopathological findings. For the purpose of this study, clinically confirmed osteomyelitis was defined as a clinical presentation consistent with bone infection, including local signs of infection such as pain, swelling, erythema, wound secretion, or exposed bone, in combination with at least one objective finding supporting osteomyelitis, such as characteristic imaging findings, intraoperative evidence of infected or necrotic bone, a positive microbiological culture from a deep tissue or bone specimen, or histopathological evidence of osteomyelitis or inflammatory changes compatible with bone infection.

Microbiological and histopathological specimens were obtained as part of the diagnostic work-up and were included in the retrospective assessment. Diagnoses were made independently by the treating physicians as part of routine clinical care; no retrospective central adjudication of the diagnoses was performed.

Both acute and chronic presentations were eligible for inclusion in order to capture the full spectrum of hand osteomyelitis treated at our institution during the study period. No recurrent episodes of osteomyelitis were identified in the study cohort; consequently, each patient contributed only one episode to the analysis.

The following variables were extracted from the electronic medical records: patient age, sex, anatomical location of osteomyelitis, underlying etiology, presence of immunosuppression like oncological disease, diabetes mellitus, HIV infection, or ongoing systemic corticosteroid therapy, microbiological findings, histopathological diagnosis, preoperative imaging modality, antibiotic treatment, duration of antibiotic therapy, number of surgical procedures, need for amputation, inflammatory laboratory parameters at admission (C-reactive protein [CRP] and leukocyte count), and length of hospital stay.

All patients received their primary treatment at our institution, and no surgical procedures performed before referral were included. All surgical interventions were performed in the operating room; bedside procedures were not performed or included in the analysis. Each separate operation was counted as one surgical procedure, including biopsy, soft-tissue revision, bone debridement, arthrotomy, partial amputation, and complete amputation. When more than one procedure was performed during the same operative session, the intervention was counted as a single surgical procedure. For the quantitative analysis, all surgical procedures were weighted equally, irrespective of their type or extent. Accordingly, the number of surgical procedures was used as a descriptive measure of treatment burden and should not be interpreted as a direct measure of disease severity.

Immunosuppression was defined as the presence of immunosuppressive medication or an underlying disease associated with impaired immune function.

Statistical analyses were performed using IBM SPSS Statistics 31 (IBM Corp., Armonk, NY, USA). Continuous variables are presented as mean values with ranges, whereas categorical variables are reported as absolute numbers and percentages. Given the small sample size, the distribution of continuous variables was assessed using the Shapiro–Wilk test, with the results interpreted cautiously due to the limited power of formal normality testing in small samples. Correlations between admission inflammatory markers (CRP and leukocyte count), number of surgical procedures, and length of hospital stay were initially assessed using Pearson’s correlation coefficient (r) to quantify linear associations between continuous variables. As some variables showed evidence of non-normality, Spearman’s rank correlation coefficient (ρ) was additionally calculated as a sensitivity analysis to assess the robustness of the observed associations. For Pearson’s correlation coefficients, 95% confidence intervals (CIs) were calculated to quantify the uncertainty of the estimates. Statistical significance was defined as a two-sided *p* value of <0.05. Owing to the small sample size, all statistical analyses were considered exploratory and descriptive, and the results were interpreted cautiously. The study was submitted to and reviewed by the local Ethics Committee (Approval No. 406/17). Due to the retrospective nature of the study, the requirement for informed consent was waived.

## 3. Results

A total of 14 patients with hand osteomyelitis were included in the analysis. The mean age was 53.7 years (range, 6–90 years). Nine patients (64.3%) were female and five (35.7%) were male. Osteomyelitis was predominantly located in the distal phalanx (13/14, 92.9%), while only one patient presented with involvement of the proximal phalanx. The mean follow-up duration was 12.6 months, with a median follow-up of 1.4 months.

The underlying causes of osteomyelitis were heterogeneous and included traumatic injuries, nail bed infections, foreign body injuries, postoperative infections, and chronic underlying diseases associated with soft tissue ulceration. Nine patients (64.3%) had relevant immunosuppression underlying condition.

**Figure 4:**
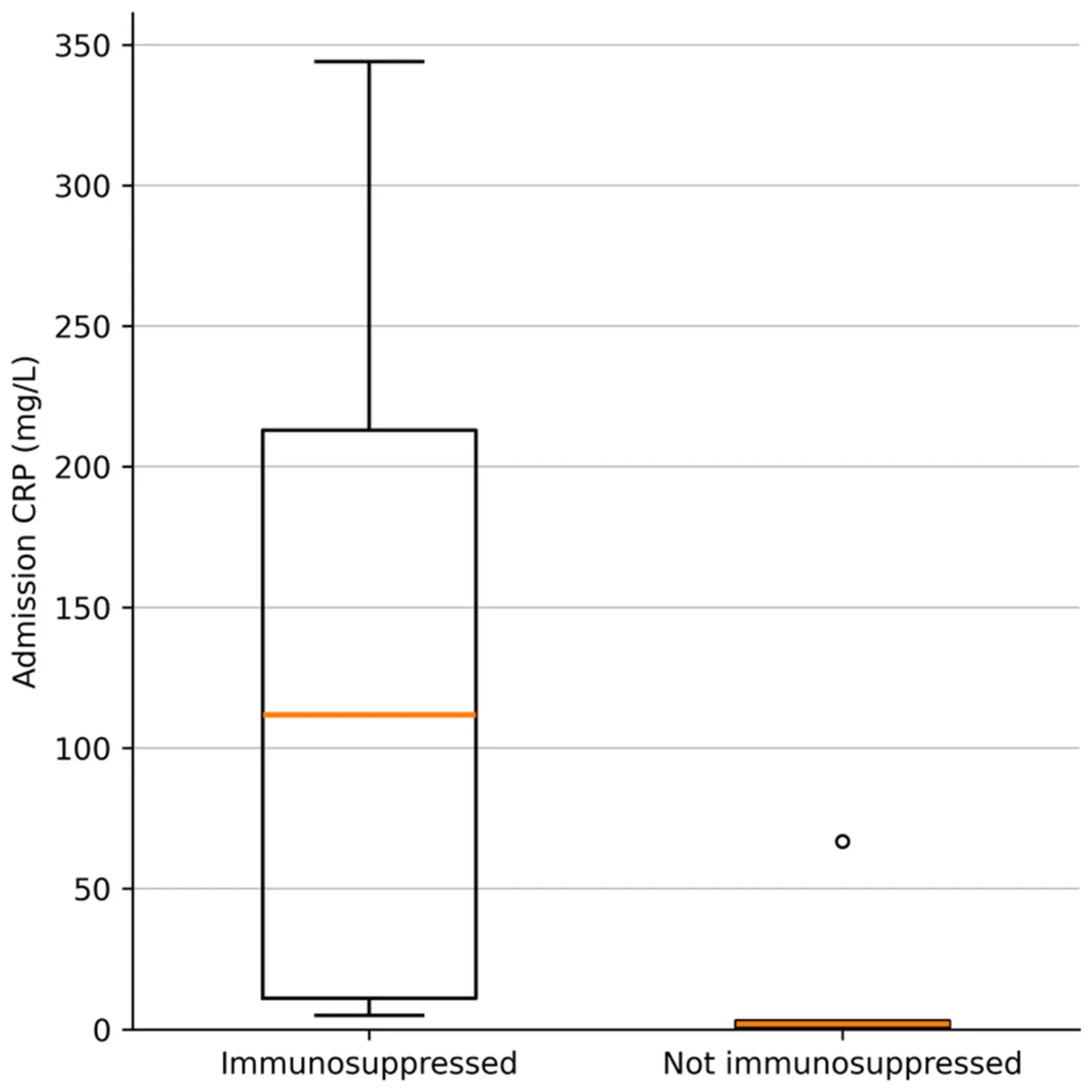
Box plot comparing admission C-reactive protein (CRP) levels between immunosuppressed and non-immunosuppressed patients. Immunosuppressed patients exhibited substantially higher CRP levels at presentation than non-immunosuppressed patients (mean 128.5 vs. 14.6 mg/L).

At admission, the median C-reactive protein (CRP) level was 38.9 mg/L (IQR 3.5–157.7), and the mean leukocyte count was 12.4 × 10^9^/L. Patients with immunosuppression presented with markedly higher inflammatory markers than those without immunosuppression (CRP: 128.5 vs. 14.6 mg/L; leukocyte count: 14.0 vs. 9.7 × 10^9^/L) (Figure 4).

Imaging consisted of magnetic resonance imaging (MRI) in seven patients, conventional radiography in five patients, and computed tomography (CT) in two patients.

Amputation was performed in 11 patients (78.6%). Of these, four patients (36.4%) underwent primary amputation because the affected digit or segment was considered non-salvageable, whereas seven patients (63.6%) underwent secondary amputation following previous surgical procedures when adequate infection control and preservation of a functional digit or segment could not be achieved. The median number of surgical procedures was 4.0 (IQR 1.0–4.8).

Microbiological analysis most frequently identified *Staphylococcus aureus*, methicillin-resistant *Staphylococcus aureus* (MRSA), and *Streptococcus pyogenes*. In addition, various Gram-negative organisms, anaerobic bacteria, and *Candida albicans* were isolated, reflecting a broad spectrum of pathogens. Patients with immunosuppression harbored a higher mean number of different bacteria than patients without immunosuppression (2.0 vs. 1.4 pathogens per patient).

Antimicrobial therapy was tailored according to microbiological findings and most commonly included cefuroxime, ampicillin/sulbactam, and clindamycin. Combination regimens with piperacillin/tazobactam, ciprofloxacin, or fluconazole were administered in selected cases. The median duration of antibiotic therapy was 35.5 days (IQR 12.5–42.0).

Histopathological examination confirmed active osteomyelitis or pronounced acute or chronic inflammatory changes in the majority of cases.

The median length of hospital stay was 12.0 days (IQR 7.0–26.3). Given the small sample size and evidence of non-normality for some variables, Spearman’s rank correlation was used as the primary measure of association. Confidence intervals for Spearman’s ρ were estimated using bootstrap resampling.

**Figure 5:**
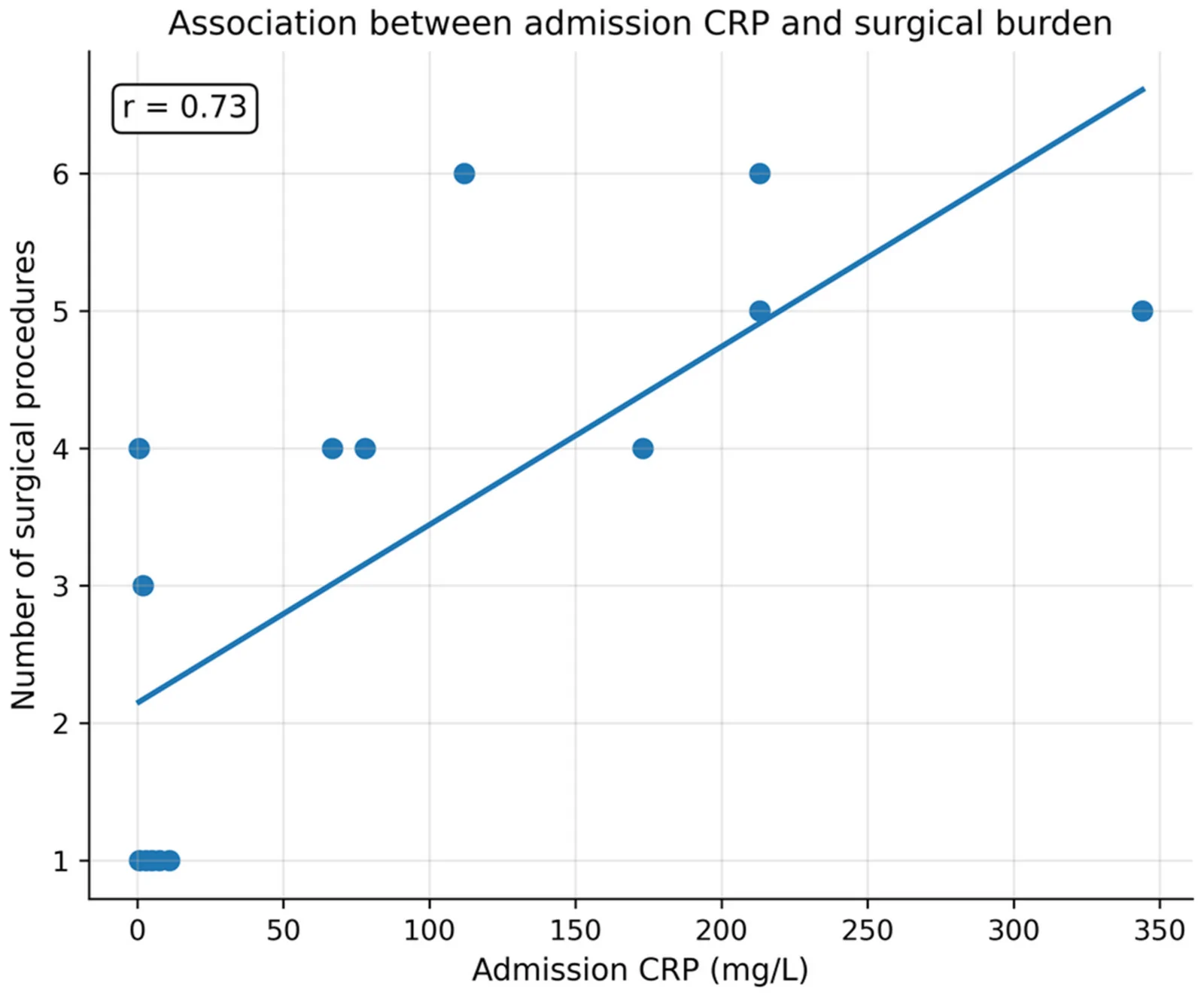
Correlation between admission C-reactive protein (CRP) levels and the number of surgical procedures. Patients presenting with higher inflammatory marker levels required more operative interventions, suggesting that admission CRP may serve as a marker of disease severity in hand osteomyelitis (r = 0.73).

Admission CRP was positively associated with length of hospital stay (Spearman ρ = 0.62, 95% bootstrap CI 0.19–0.83, *p* = 0.019) and with the number of surgical procedures (ρ = 0.72, 95% bootstrap CI 0.27–0.92, *p* = 0.004) (Figure 5). The number of surgical procedures was also positively associated with length of hospital stay (ρ = 0.69, 95% bootstrap CI 0.15–0.97, *p* = 0.007). These findings indicate that admission CRP was associated with treatment burden in this exploratory cohort.

In contrast, admission leukocyte count showed weaker associations with length of hospital stay (ρ = 0.17, 95% bootstrap CI −0.35–0.66, *p* = 0.572) and the number of surgical procedures (ρ = 0.25, 95% bootstrap CI −0.30–0.66, *p* = 0.383). Admission CRP and leukocyte count were positively correlated (ρ = 0.62, 95% bootstrap CI 0.09–0.92, *p* = 0.017).

Pearson correlation analyses were performed as sensitivity analyses and showed a broadly similar pattern. Admission CRP was correlated with length of hospital stay (Pearson r = 0.61, 95% CI 0.12–0.86, *p* = 0.020) and with the number of surgical procedures (r = 0.73, 95% CI 0.32–0.91, *p* = 0.003). The number of surgical procedures was correlated with length of hospital stay (r = 0.63, 95% CI 0.14–0.87, *p* = 0.016). Admission leukocyte counts showed weaker correlations with length of hospital stay (r = 0.36, 95% CI −0.21–0.75, *p* = 0.207) and the number of surgical procedures (r = 0.29, 95% CI −0.28–0.71, *p* = 0.312). Admission CRP and leukocyte count were positively correlated (r = 0.71, 95% CI 0.29–0.90, *p* = 0.004).

Patients with immunosuppression exhibited higher inflammatory markers at admission than those without immunosuppression. The mean admission CRP level was 128.5 mg/L in immunosuppressed patients compared with 14.6 mg/L in non-immunosuppressed patients. Given the small sample size, these findings should be considered descriptive.

**Figure 6:**
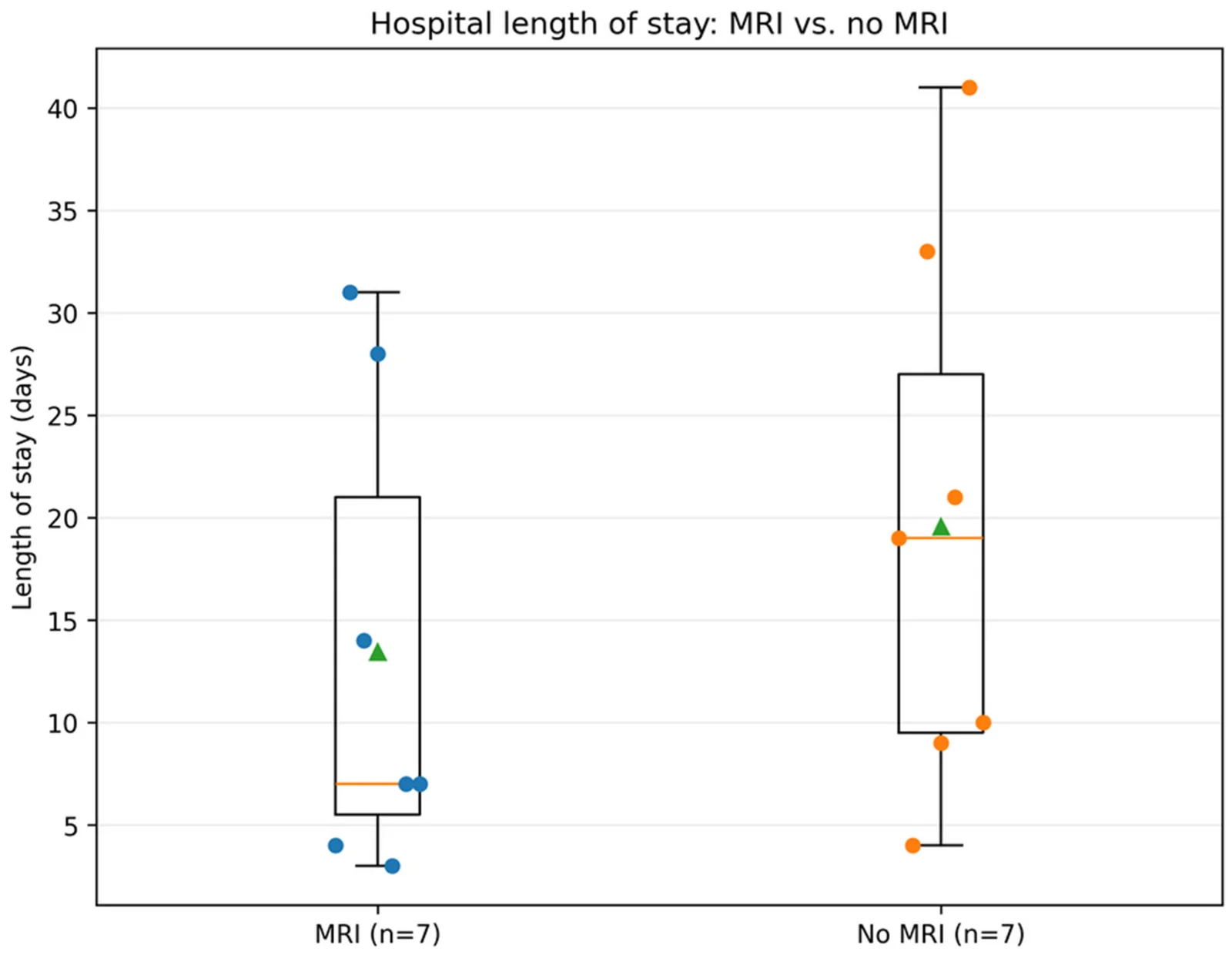
Box plot comparing the length of hospital stay between patients who underwent preoperative magnetic resonance imaging (MRI) and those who did not. Patients who underwent preoperative MRI had a shorter mean hospital stay than patients without MRI (13.4 vs. 19.6 days).

Patients who underwent preoperative MRI had a shorter mean length of hospital stay than those who did not undergo MRI (13.4 vs. 19.6 days). Given the small sample size and the observational nature of the analysis, this finding should be interpreted as descriptive and does not establish a causal effect of preoperative MRI on length of hospital stay (Figure 6).

## 4. Discussion

Hand osteomyelitis remains a rare but challenging condition because of its heterogeneous etiology, delayed diagnosis, and the absence of standardized treatment algorithms. The recently published systematic review by Dargan et al., which analyzed 666 reported cases, highlights the low level of evidence currently available and emphasizes that treatment recommendations are largely based on retrospective case series rather than prospective studies [1].

**Figure 7:**
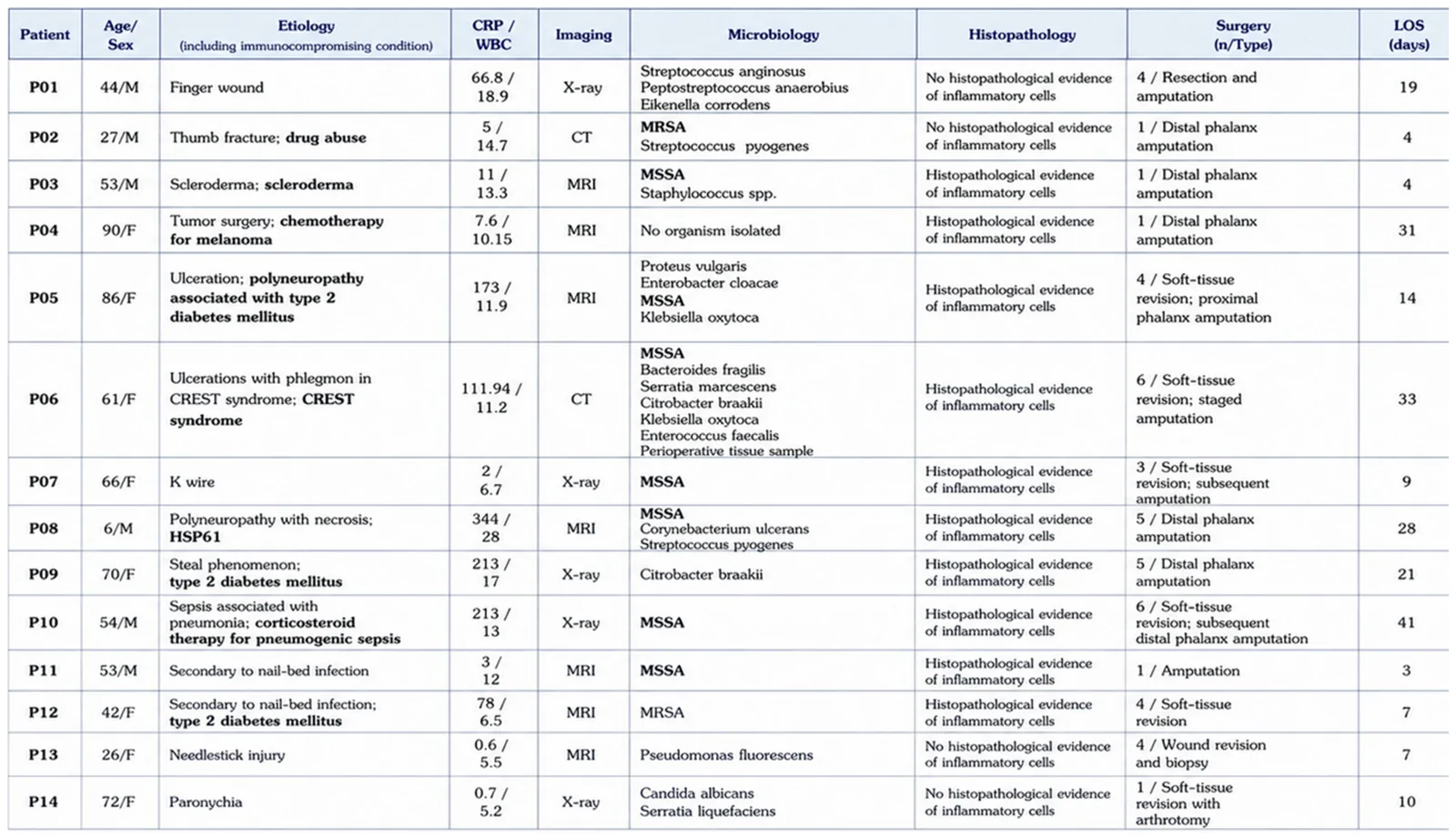
Patient characteristics and clinical findings of the study cohort. Individual patient data are presented for all 14 patients with hand osteomyelitis, including age and sex, infection etiology, admission C-reactive protein (CRP) and white blood cell count (WBC), imaging modality, microbiological findings, histopathological findings, number and type of surgical procedures, and hospital length of stay (LOS). MRSA, methicillin-resistant *Staphylococcus aureus*; CT, computed tomography; MRI, magnetic resonance imaging.

The present study reflects the heterogeneous nature of hand osteomyelitis described in previous reports. Most infections involved the distal phalanx and resulted from trauma, nail bed infections, foreign body injuries, or postoperative complications (Figure 7). These findings are consistent with published series demonstrating that direct inoculation following trauma or surgery represents the predominant mechanism of infection in the hand [1,11].

Nearly two-thirds of our patients presented with relevant immunosuppression or immunocompromising comorbidities. Immunosuppressed patients demonstrated substantially higher CRP values at admission and a higher number of isolated pathogens compared with immunocompetent individuals. Although statistical significance cannot be inferred because of the limited cohort size, these findings support the concept that impaired host immunity is associated with more severe and microbiologically complex infections [1,2].

The microbiological spectrum in our cohort was dominated by *Staphylococcus aureus*, MRSA, and *Streptococcus pyogenes*, whereas Gram-negative bacteria, anaerobes, and fungal pathogens were isolated less frequently. This distribution is consistent with previous reports identifying *Staphylococcus aureus* as the predominant pathogen in hand osteomyelitis while emphasizing that polymicrobial infections are common, particularly in patients with diabetes mellitus, renal failure, vascular disease, or bite injuries [1,12,13].

Patients with immunosuppression harbored a greater number of microorganisms than non-immunosuppressed patients (2.0 vs. 1.4 pathogens per patient). This observation is clinically relevant because polymicrobial infections may require broader empiric antimicrobial coverage until microbiological culture results become available [1,12].

In the present series, histopathological examination confirmed osteomyelitis in the majority of patients. Bone biopsy combined with microbiological culture remains the diagnostic gold standard, allowing confirmation of infection, assessment of chronicity, and exclusion of differential diagnoses such as neoplasia or granulomatous disease [2,4,5].

Our data demonstrated that admission CRP correlated moderately with the length of hospital stay and even more strongly with the number of surgical procedures. In contrast, leukocyte count showed only weak correlations with both outcomes. These findings suggest that CRP may better reflect disease severity than leukocyte count in patients with hand osteomyelitis [14].

This observation agrees with the retrospective study by Dargan et al., who demonstrated that CRP is considerably more sensitive than white blood cell count for diagnosing hand osteomyelitis and that increasing CRP values during follow-up were associated with an increased risk of amputation [1,14].

Similarly, systematic reviews investigating osteomyelitis in diabetic foot infections have demonstrated that CRP is a more reliable inflammatory biomarker than leukocyte count, although laboratory parameters alone are insufficient for establishing the diagnosis [9].

**Figure 8:**
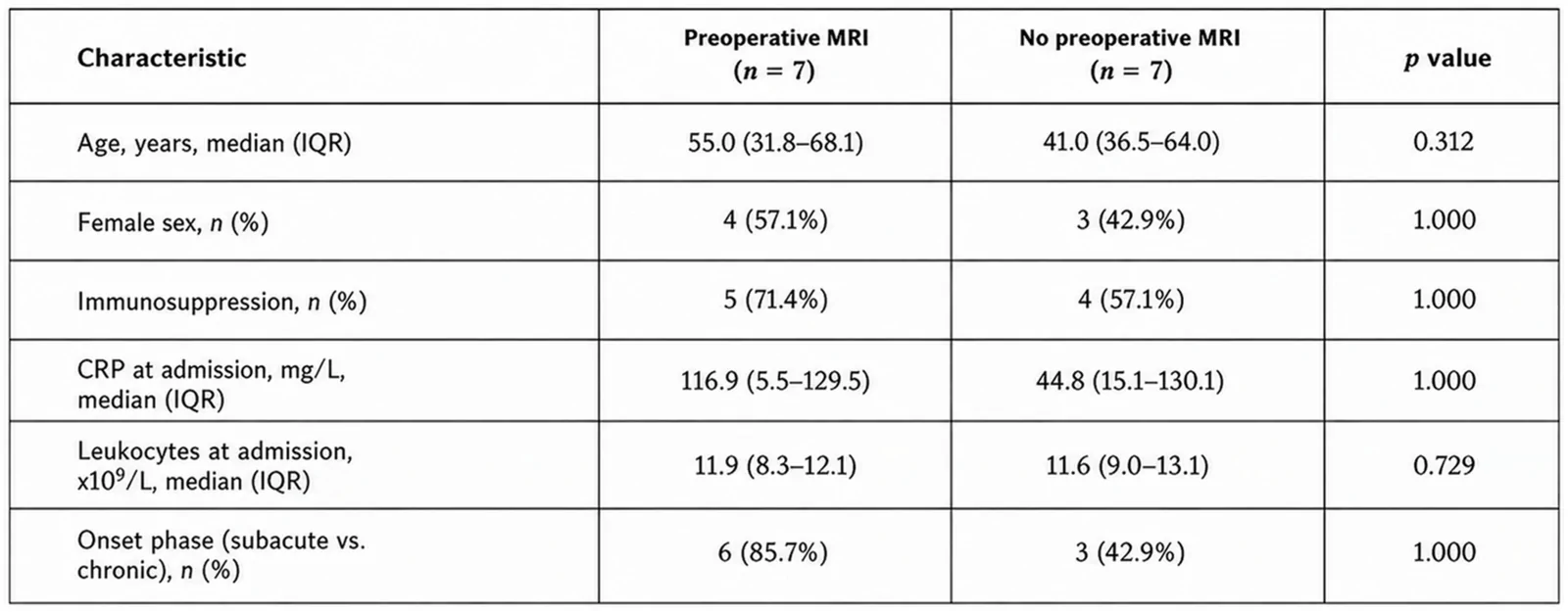
Baseline characteristics according to preoperative MRI status. Comparison of baseline clinical and laboratory characteristics between patients who underwent preoperative magnetic resonance imaging (MRI) and those who did not. Data are presented as median (interquartile range [IQR]) or number (percentage). Continuous variables were compared using the Mann–Whitney U test, and categorical variables using Fisher’s exact test. All *p* values are two-sided and should be interpreted as exploratory given the small sample size.

An interesting finding of our study was that patients who underwent an MRI had a shorter hospital stay than patients who did not receive MRI (Figure 8). Although this association should not be interpreted as causal, MRI may facilitate earlier diagnosis and more accurate surgical planning. Current recommendations consider MRI to be the imaging modality of choice whenever diagnostic uncertainty exists or early osteomyelitis is suspected [1,7,8,9].

The overall amputation rate of 78.6% appears high compared with many published case series. However, our institution serves as a tertiary referral center, and a substantial proportion of patients presented with advanced infection, immunosuppression, or severe comorbidities requiring definitive surgical treatment. Delayed presentation has repeatedly been identified as one of the strongest predictors of amputation in hand osteomyelitis [1,3,15]. Furthermore, the extent of osseous involvement and the physiological host status are well-recognized determinants of treatment strategy and outcome in osteomyelitis, as reflected by the Cierny–Mader classification [16].

The average duration of antibiotic treatment in our cohort was approximately four weeks, which is consistent with current recommendations advocating four to six weeks of pathogen-directed antimicrobial therapy following adequate surgical debridement. Following complete amputation of the infected bone, substantially shorter antibiotic courses may be sufficient [1,17,18,19].

Recent evidence has challenged the traditional paradigm that prolonged intravenous antibiotic therapy is mandatory for osteomyelitis. The OVIVA trial demonstrated that oral antibiotic therapy is non-inferior to intravenous treatment for bone and joint infections after the acute septic phase, thereby supporting an earlier transition to oral treatment in appropriately selected patients. Although only a minority of patients in the OVIVA trial had hand osteomyelitis, these findings have influenced contemporary treatment recommendations [5,10].

The present study has several limitations. First, its retrospective design is inherently associated with selection bias and incomplete documentation. Second, the small sample size precluded multivariable statistical analyses and limits the generalizability of our findings. Third, the heterogeneity of underlying etiologies, pathogens, and comorbidities reflects routine clinical practice but complicates subgroup analyses. Finally, functional outcomes after treatment could not be assessed because long-term follow-up data were not consistently available.

Despite these limitations, our study contributes additional data to the limited literature on hand osteomyelitis. In particular, the observed association between elevated admission CRP, increased surgical burden, and prolonged hospitalization suggests that CRP may represent a useful marker for estimating disease severity. Furthermore, our findings underline the importance of early diagnosis, prompt surgical debridement with microbiological sampling, and individualized antimicrobial therapy, particularly in immunocompromised patients who appear to be at risk for more extensive and polymicrobial infections [1,2,14]. Furthermore, multiple exploratory statistical comparisons were performed without adjustment for multiple testing, increasing the risk of type I error and the possibility that some statistically significant findings may have occurred by chance. In addition, no multivariable analysis was performed due to the limited sample size. This represents an important limitation, as both length of hospital stay and the number of surgical procedures may be influenced by several clinical factors, including disease severity, comorbidities, immunosuppression, referral pathway, timing of diagnosis, and treatment-related factors. Consequently, potential confounding cannot be excluded, and the observed associations, particularly those involving admission CRP and preoperative MRI, should not be interpreted as independent or causal effects. Given these limitations and the exploratory nature of the study, the findings should be interpreted cautiously and require confirmation in larger prospective cohorts using appropriately adjusted multivariable analyses.

## 5. Conclusions

Hand osteomyelitis remains a challenging condition in which timely diagnosis and appropriate surgical management are important. In this retrospective cohort, elevated admission CRP levels were associated with a higher number of surgical procedures and a longer hospital stay. Admission CRP was associated with treatment burden in this exploratory cohort; however, this association requires confirmation in larger cohorts. Patients who underwent preoperative MRI had a shorter length of hospital stay, indicating a possible association between early advanced imaging and the subsequent clinical course. However, given the retrospective study design, no conclusions regarding a causal effect of MRI on length of stay can be drawn. MRI use was associated with a shorter unadjusted hospital length of stay in this small cohort; however, selection and confounding prevent causal interpretation. Immunosuppressed patients showed higher inflammatory markers and a tendency toward polymicrobial infections, which may warrant particular attention to microbiological assessment and individualized antimicrobial management in this patient population. Given the small sample size and retrospective design, these findings should be considered exploratory. Larger prospective studies are needed to determine the prognostic value of admission CRP and to further clarify the potential role of preoperative MRI in the management of hand osteomyelitis.

## Data Availability

The data analyzed in this study were retrospectively obtained from routine clinical diagnostics and are not publicly available due to data protection regulations. However, the data may be made available by the corresponding author upon reasonable request.

## References

[1] Dargan D., Wyman M., Bhoora M., Ronan D., Baker M., Partridge D., Caddick J., Giblin V. (2025). Hand Osteomyelitis: A Systematic Review of the Literature and Recommendations for Diagnosis and Management. Hand.

[2] Hatzenbuehler J., Pulling T.J. (2011). Diagnosis and management of osteomyelitis. Am. Fam. Physician.

[3] Calhoun J.H., Manring M.M. (2005). Adult osteomyelitis. Infect. Dis. Clin. N. Am..

[4] Lew D.P., Waldvogel F.A. (2004). Osteomyelitis. Lancet.

[5] Henry M., Lundy F.H. (2021). Oral Antibiotic Management of Acute Osteomyelitis of the Hand: Outcomes and Cost Comparison to Standard Intravenous Regimen. Hand.

[6] Shirtliff M.E., Mader J.T. (2002). Acute septic arthritis. Clin. Microbiol. Rev..

[7] Pierce J.L., Perry M.T., Wessell D.E., Lenchik L., Ahlawat S., Baker J.C., Banks J., Caracciolo J.T., DeGeorge K.C., Demertzis J.L. (2022). ACR Appropriateness Criteria^®^ Suspected Osteomyelitis, Septic Arthritis, or Soft Tissue Infection (Excluding Spine and Diabetic Foot): 2022 Update. J. Am. Coll. Radiol..

[8] Termaat M.F., Raijmakers P.G.H.M., Scholten H.J., Bakker F.C., Patka P., Haarman H.J.T.M. (2005). The accuracy of diagnostic imaging for the assessment of chronic osteomyelitis: A systematic review and meta-analysis. J. Bone Jt. Surg.-Am..

[9] Ansert E.A., Tarricone A.N., Coye T.L., Crisologo P.A., Truong D., Suludere M.A., Lavery L.A. (2024). Update of biomarkers to diagnose diabetic foot osteomyelitis: A meta-analysis and systematic review. Wound Repair Regen..

[10] Li H.-K., Rombach I., Zambellas R., Walker A.S., McNally M.A., Atkins B.L., Lipsky B.A., Hughes H.C., Bose D., Kümin M. (2019). Oral versus Intravenous Antibiotics for Bone and Joint Infection. N. Engl. J. Med..

[11] Gundlach B.K., Sasor S.E., Chung K.C. (2020). Hand Infections: Epidemiology and Public Health Burden. Hand Clin..

[12] Brook I. (2008). Microbiology and management of soft tissue and muscle infections. Int. J. Surg..

[13] Fuchsjäger N., Winterleitner H., Krause R., Feierl G., Koch H. (2019). Susceptibility of microorganisms causing acute hand infections. PLoS ONE.

[14] Michail M., Jude E., Liaskos C., Karamagiolis S., Makrilakis K., Dimitroulis D., Michail O., Tentolouris N. (2013). The performance of serum inflammatory markers for the diagnosis and follow-up of patients with osteomyelitis. Int. J. Low. Extrem. Wounds.

[15] Clark D.C. (2003). Common acute hand infections. Am. Fam. Physician.

[16] Cierny G., Mader J.T., Penninck J.J. (2003). A clinical staging system for adult osteomyelitis. Clin. Orthop. Relat. Res..

[17] Flevas D.A., Syngouna S., Fandridis E., Tsiodras S., Mavrogenis A.F. (2019). Infections of the hand: An overview. EFORT Open Rev..

[18] Osterman M., Draeger R., Stern P. (2014). Acute hand infections. J. Hand Surg. Am..

[19] Rerucha C.M., Ewing J.T., Oppenlander K.E., Cowan W.C. (2019). Acute Hand Infections. Am. Fam. Physician.

